# The 3D characteristics of nystagmus in posterior semicircular canal benign paroxysmal positional vertigo

**DOI:** 10.3389/fnins.2022.988733

**Published:** 2022-12-13

**Authors:** Yao Liu, Xueqing Zhang, Qiaomei Deng, Qiang Liu, Chao Wen, Wei Wang, Taisheng Chen

**Affiliations:** ^1^Department of Otorhinolaryngology Head and Neck Surgery, Tianjin First Central Hospital, Tianjin, China; ^2^Institute of Otolaryngology of Tianjin, Tianjin, China; ^3^Key Laboratory of Auditory Speech and Balance Medicine, Tianjin, China; ^4^Key Medical Discipline of Tianjin (Otolaryngology), Tianjin, China; ^5^Quality Control Centre of Otolaryngology, Tianjin, China

**Keywords:** benign positional paroxysmal vertigo, three-dimensional video nystagmography, canalithiasis, semicircular canal, Ewald’s law

## Abstract

**Objective:**

The aim of this study was to observe the 3-dimensional (3D; horizontal, vertical, and torsional) characteristics of nystagmus in patients with posterior semicircular canal canalithiasis (PSC-can)–related benign paroxysmal positional vertigo (BPPV) and investigate its correlation with Ewald’s.

**Methods:**

In all, 84 patients with PSC-can were enrolled. The latency, duration, direction, and slow-phase velocity induced by the Dix-Hallpike test in the head-hanging and sitting positions were recorded using 3D video nystagmography (3D-VNG). The characteristics of the horizontal, vertical, and torsional components of nystagmus were quantitatively analyzed.

**Results:**

3D-VNG showed that the fast phase of the vertical components and torsional components of left and right ear PSC-can as induced by the head-hanging position of the Dix-Hallpike test were upward, clockwise and counterclockwise, and horizontal components were mainly contralateral. The median slow-phase velocity of each of the three components for consecutive 5 s was 26.3°/s (12.3–45.8), 25.0°/s (15.7–38.9), and 9.2°/s (4.9–13.7). When patients were returned to the sitting position, the fast phase of the vertical and torsional components of nystagmus was reversed. Only 54 patients had horizontal components of nystagmus, and 32 of them remained in the same direction. The median slow-phase velocity of the three components for consecutive 5 s was 9.4°/s (6.0–11.7), 6.8°/s (4.5–11.8), and 4.9°/s (2.8–8.0). The ratios of the slow-phase velocity of the horizontal, vertical, and torsional components of the head-hanging position to the sitting position were close to 1.85 (1.0–6.6), 3.7 (1.9–6.6), and 5.1 (2.6–11.3). The ratios of the slow-phase velocity of the vertical to horizontal component, the torsional to horizontal component, and the vertical to torsional component of the head-hanging position were close to 3.3 (1.7–7.6), 3.9 (1.8–7.6), and 1.0 (0.5–1.8). The ratios of the slow-phase velocity of the vertical to horizontal component, the torsional to horizontal component, and the vertical to torsional component of the sitting position were close to 2.1 (1.1–6.8), 1.5 (1.0–3.8), and 1.2 (0.8–2.8).

**Conclusion:**

There were three components of nystagmus induced by the Dix-Hallpike test in patients with PSC-can. The vertical component was the strongest and the horizontal component was the weakest. The 3D characteristics of nystagmus were consistent with those of physiological nystagmus associated with the same PSC with a single-factor stimulus, in accordance with Ewald’s law.

## Introduction

Benign paroxysmal positional vertigo (BPPV) is the most common peripheral vestibular disorder, accounting for 17–42% of patients with vertigo (Power et al., 2020; Kim et al., 2021; Palmeri and Kumar, 2022). Posterior semicircular canal (PSC) involvement is common (Shigeno and Kitaoka, 2020). BPPV is a common peripheral vestibular disease, whose basic pathologic process is caused by the migration of otoconia from the utricular macula to the semicircular canals (Bhattacharyya et al., 2017). The canalith repositioning procedure has confirmed this pathological theory. In 1824 (Shimizu, 2000) and 1892 (Baloh and Honrubia, 1979), Flourens and Ewald explored the physiological mechanism of the vestibular semicircle from the 2 levels of the phenomenon and mechanism, respectively, through animal physiological models (Yang et al., 2017). Their findings formed the basis of Flouren’s law and Ewald’s law, Ewald’s Law perfected Flourens Law from mechanism and phenomenon, and contributed greatly to the understanding of human vestibular physiology and pathology (Baloh et al., 1977). Whether this law is completely consistent with human semicircular canal physiology has remained difficult to verify in the human body for quite some time. In the horizontal semicircular canal canalithiasis/cupulolithiasis, posterior/anterior semicircular canal canalithiasis/cupulolithiasis, or in the roll test left/right head turning and Dix-Hallpike Test head-hanging/sitting position, otoliths are diagnosed and localized by inducing excitatory or inhibitory effects under the action of a single stimulant, a specific otoconia. Therefore, similar to horizontal semicircular canal BPPV (HSC-BPPV) (Zhang et al., 2021), PSC-BPPV also objectively provides a physiologically effective model for understanding and studying the response effect of single semicircular canal stimulation under the action of a “single factor.” BPPV is not so much a disease as a physiological reaction of the vestibular semicircular canal to physiological properties. Through BPPV and positional testing, Flourens and Ewald’s laws from animal physiological models were finally interpreted in relation to the human body. Therefore, BPPV is also the best way to understand human semicircular canal physiology and the semicircular canal nystagmus effect, helping to analyze the functional characteristics of the human semicircular canal and its nystagmus effect.

Currently, the diagnosis of PSC canalithiasis (PSC-can) is mainly based on the naked eye observation of the vertical and torsional components of nystagmus induced by the Dix-Hallpike test (Brevern et al., 2017). The application of traditional videonystagmography (VNG) makes the analysis of BPPV nystagmus and the localization of otolith localization from subjective to objective, but the torsion component of PSC could not be observed (Xu et al., 2019; Zhang et al., 2021). Three dimensional (3D)-VNG can accurately quantify the horizontal, vertical, and torsional components of PSC-can nystagmus, but there were few quantitative studies of 3D-VNG for PSC-can (Fetter and Sievering, 1995a,b; Imai et al., 2009; Ichijo, 2013). In this study, 3D-VNG was used to record and quantitatively analyze the horizontal, vertical, and torsional components of nystagmus induced by the Dix-Hallpike test in patients with PSC-can. Furthermore, we explored the correlation between the nystagmus of PSC-can and Ewald’s law. We hope the findings can contribute to the study of the physiological characteristics of the posterior semicircular canal and make the diagnosis of PSC-can more objective.

## Materials and methods

### Participants

This was an observational study involving the assessment of 84 patients with PSC-can who were examined at the Ear, Nose, and Throat (ENT) Department of the Tianjin First Central Hospital between September 2021 and January 2022. All of the patients had successfully been treated with the canalith repositioning procedure (CRP), and all provided informed consent prior to their inclusion in the study. The study procedures were approved by the Ethics Committee of the Tianjin First Central Hospital (number: 2020N118KY).

The inclusion criteria were the following: a history of positional vertigo as a predominant symptom, accompanied by autonomic symptoms such as nausea and vomiting; and a diagnosis of PSC-can according to the Bárány guidelines (Brevern et al., 2017) with successful repositioning. Meanwhile, the exclusion criteria were the following: horizontal semicircular canal benign paroxysmal positional vertigo (HSC-BPPV), anterior semicircular canal benign paroxysmal positional vertigo (ASC-BPPV), multiple-canal BPPV, or cupulolithiasis; and other types of peripheral and central vertigo, as identified on neurological and imaging examinations. Vestibular migraine was identified and excluded according to the International vestibular Migraine Guidelines.

### Methods

The Dix-Hallpike test was performed to diagnose PSC-can by 3D-VNG (VertiGoggles-M, ZEHNIT Medical Technology-VNG-II, Shanghai, China). The directional characteristics of the torsional and vertical components of nystagmus induced in the left and right head-hanging positions and the sitting position were observed by monitor. The latency, duration, direction, and slow-phase velocity of the horizontal, vertical, and torsional components were simultaneously recorded by 3D-VNG. During the Dix-Hallpike test, the patient’s eyes should remain directly in front of the face (i.e., the eyes should remain in place at all times). For the Dix-Hallpike test, the patient’s eyes should be kept directly in front of the face (i.e., in place at all times).

**FIGURE 1:**
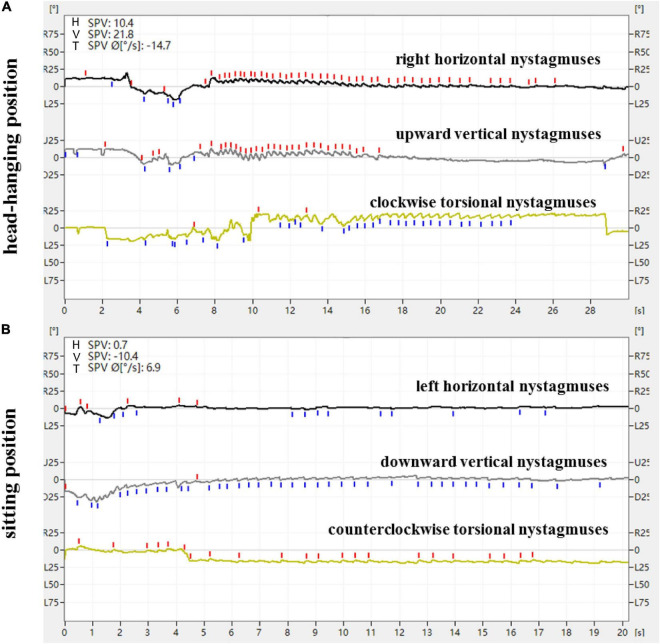
Expression of nystagmus on the 3D-VNG by Dix-Hallpike test of a patient with L-PSC-can. **(A)** At the head-hanging position, the right horizontal nystagmuses were recorded as upward fast-phase nystagmus curves, the upward vertical nystagmuses were recorded as upward fast-phase nystagmus curves, and the clockwise torsional nystagmuses were recorded as downward fast-phase nystagmus curves. **(B)** The direction of nystagmus was reversed when the head was recovered from the head-hanging position to the sitting position.

**FIGURE 2:**
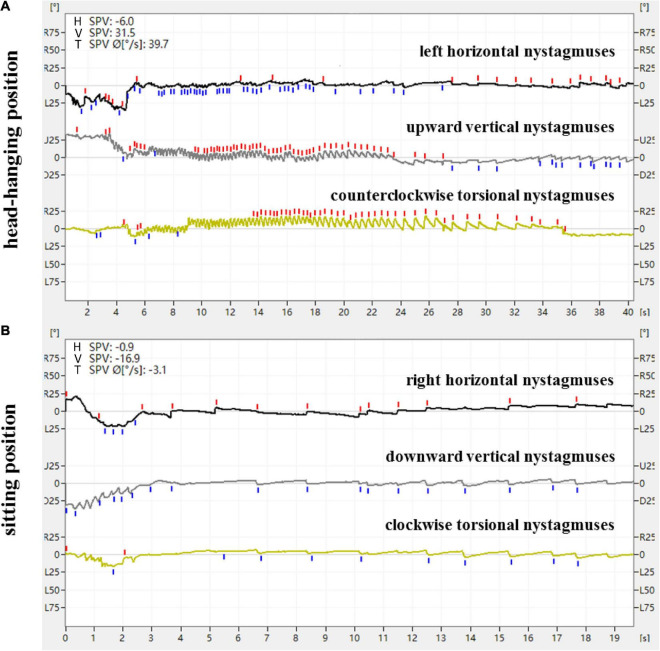
Expression of nystagmus on the 3D-VNG by Dix-Hallpike test of a patient with R-PSC-can. **(A)** At the head-hanging position, the left horizontal nystagmuses were recorded as downward fast-phase nystagmus curves, the upward vertical nystagmuses were recorded as upward fast-phase nystagmus curves, and the counterclockwise torsional nystagmuses were recorded as upward fast-phase nystagmus curves. **(B)** The direction of nystagmus was reversed when the head was recovered from the head-hanging position to the sitting position.

Based on the Bárány diagnosis and treatment guidelines (Brevern et al., 2015, 2017), from a doctor’s perspective, using the apex of the eyeball as a marker and the patient as a reference, the monitor observed vertical upward nystagmus with a torsional component (i.e., the vertical component was marked by upward movement, and the torsional component was marked by the apex of the eyeball turning to the lower ear) when the affected ear at the head-hanging position was turned to the ground in the Dix-Hallpike test. That is, right (R)-PSC-can induced right torsional nystagmus (counterclockwise), and left (L)-PSC-can induced left torsional nystagmus (clockwise). After the patient had returned to the sitting position, the fast phase of nystagmus was reversed. Therefore, on the 3D-VNG diagram, the left/right horizontal nystagmuses were recorded as downward/upward fast phase nystagmus curves, the downward/upward vertical nystagmuses were recorded in the same direction as the real nystagmus, and the clockwise/counterclockwise torsional nystagmuses were recorded as downward/upward fast phase nystagmus curves (Figures 1, 2). Latency time was recorded as the period from the end of a head turn (in the head-hanging or sitting position) to the onset of continuous nystagmus. Duration was recorded as the period of continuous nystagmus, from its onset to the end. Based on the nystagmus duration of <10 s for some patients, the analysis of slow-phase velocity included the average slow-phase velocity of 5 s, 10 s, and the entire duration. The roll test was used to exclude HSC-BPPV and multiple-canal BPPV.

### Analysis

The parameters of torsional nystagmus induced by the Dix-Hallpike test were compared within and between the groups. Continuous variables are expressed as the means ± SD or medians (interquartile ranges [IQR], Q1-Q3); categorical variables are expressed as frequencies and percentages. Comparisons of means between the two groups were performed using the independent *t*-test or the Mann-Whitney *U* test as appropriate. The chi-square test was used to compare proportions in demographics. *P*-values < 0.05 were considered statistically significant. SPSS Statistics 25 (IBM Corp., Armonk, NY, USA) was used for the statistical analyses, and GraphPad Prism 5 (GraphPad, San Diego, CA, USA) and R scripts were used to generate the figures.

## Results

### General demographic characteristics of subjects

In all, 84 patients with PSC-can were enrolled in this study, including 29 males and 55 females (age range 25–80 years; mean age 54.7 years). There were 25 patients with L-PSC-can and 59 patients with R-PSC-can. Demographic data for the BPPV patients are summarized in Table 1. The vertigo symptoms of all patients were relieved or disappeared after CRP treatment (Epley, 1992).

**TABLE 1:** Demographic features of subjects in the patients with PSC-can.

Group feature	Total	PSC-can		*P*-value
		L-PSC-can	R-PSC-can	
Number	84	25	59	
Age (years) ($\bar{\text{X}}$ ± s)	54.7 ± 13.2	55.4 ± 12.8	54.4 ± 13.4	0.741
Sex (M:F)	29:55	9:16	20:39	0.853

L-PSC-can, left posterior semicircular canal canalithiasis; R-PSC-can, right posterior semicircular canal canalithiasis; M, male; F, female.

### The characteristics of nystagmus in patients with PSC-can according to the Dix-Hallpike test

From a doctor’s perspective, with the patient used as the reference and the apex of the eyeball as the marker, the directional characteristics of nystagmus induced by the Dix-Hallpike test in the 84 patients with PSC-can were recorded by nystagmus monitoring and 3D-VNG diagram observation. When the affected ear was tilted to the ground in the head-hanging position of the Dix-Hallpike test, the nystagmus monitors could observe torsional vertical jerk nystagmus (the torsional component turned to the downward ear position, and the vertical component turned to the upward-forehead position), with the duration of nystagmus being <1 min. The fast phase of the torsional component of L-PSC-can and R-PSC-can at the head-hanging position was clockwise and counterclockwise, respectively. The fast phase of nystagmus was reversed when the head was recovered from the head-hanging position to the sitting position, but in only one case did the torsional component remain in the same direction. Under 3D-VNG, the vertical component of nystagmus was recorded, and the clockwise/counterclockwise torsional nystagmuses were recorded as downward/upward fast-phase nystagmus curves. In addition, the horizontal component of nystagmus was also recorded by 3D-VNG in 73 patients with PSC-can, with 63 cases in the contralateral and 10 cases in the ipsilateral (Figures 1, 2). The fast phase of nystagmus was reversed when the patients were returned to the sitting position. The vertical component of nystagmus was observed in 74 patients, the torsional component of nystagmus was observed in 62 patients, and the horizontal component of nystagmus was observed in 54 patients (32 cases were contralateral and 22 cases were ipsilateral).

Under 3D-VNG, 84 patients with PSC-can in the head-hanging position of the Dix-Hallpike test were observed. The median latency and duration of the horizontal component of nystagmus induced by the head-hanging position were 2.0 s (1.0–4.0) and 14.0 s (10.0–21.0), those of the vertical component of nystagmus were 2.0 s (1.0–4.0) and 15.0 s (10.0–19.8), and those the torsional component of nystagmus were 2.0 s (1.0–3.0) and 15.5 s (12.0–21.8), respectively. The median slow-phase velocity of the horizontal component at the entire duration, at the strongest 10 and 5 s were 6.6°/s (3.7–9.4), 7.7°/s (4.0–11), and 9.2°/s (4.9–13.7); those of the vertical component were 19.9°/s (9.8–30.4), 24.5°/s (11.8–36.9), and 26.3°/s (12.3–45.8); and those of the torsion component were 18.3°/s (12.3–26.3), 20.7°/s (13.6–31.6), and 25.0°/s (15.7–38.9), respectively (Table 2). When patients were returned to the sitting position from the head-hanging position, the median latency and duration of the horizontal component of nystagmus were 1.0 s (1.0–2.0) and 15.5 s (12.0–19.3), those of the vertical component of nystagmus were 1.0 s (0.5–2.0) and 16.0 s (11.5–22.3), and those of the torsional component of nystagmus were 1.0 s (0.5–2.0) and 15.0 s (10.0–20.0), respectively. The median slow-phase velocity of the horizontal component at the entire duration, at the strongest 10 and 5 s were 3.5°/s (1.8–4.6), 4.2°/s (2.5–6.6), and 4.9°/s (2.8–8.0); that of the vertical component of nystagmus was 7.2°/s (5.2–9.1), 8.4°/s (6.0–10.5), and 9.4°/s (6.0–11.7); and that of the torsion component of nystagmus was 5.0°/s (3.8–8.9), 5.9°/s (4.0–9.6), and 6.8°/s (4.5–11.8) (Table 3).

**TABLE 2:** Nystagmus characteristics of the head-hanging position in 84 patients with PSC-can.

		Median (IQR)		
		Horizontal nystagmus	Vertical nystagmus	Torsional nystagmus
Number		73	84	84
Latency (s)		2.0 (1.0–4.0)	2.0 (1.0–4.0)	2.0 (1.0–3.0)
Duration (s)		14.0 (10.0–21.0)	15.0 (10.0–19.8)	15.5 (12.0–21.8)
Direction	L-PSC-can	Right (18), Left (2)	Upward	Clockwise
	R-PSC-can	Right (8), Left (45)	Upward	Counterclockwise
Slow-phase velocity (°/s)	Total	6.6 (3.7–9.4) (73)	19.9 (9.8–30.4) (84)	18.3 (12.3–26.3) (84)
	≥10 s	7.7 (4.0–11) (65)	24.5 (11.8–36.9) (77)	20.7 (13.6–31.6) (79)
	≥5 s	9.2 (4.9–13.7) (72)	26.3 (12.3–45.8) (83)	25.0 (15.7–38.9) (84)

**TABLE 3:** Nystagmus characteristics of the sitting position in 84 patients with PSC-can.

		Median (IQR)		
		Horizontal nystagmus	Vertical nystagmus	Torsional nystagmus
Number		54	74	62
Latency (s)		1.0 (1.0–2.0)	1.0 (0.5–2.0)	1.0 (0.5–2.0)
Duration (s)		15.5 (12.0–19.3)	16.0 (11.5–22.3)	15.0 (10.0–20.0)
Direction	L-PSC-can	Left (4), Right (13)	Downward	Clockwise (1), Counterclockwise (15)
	R-PSC-can	Left (19), Right (18)	Downward	Clockwise (46)
Slow-phase velocity (°/s)	Total	3.5 (1.8–4.6) (54)	7.2 (5.2–9.1) (74)	5.0 (3.8–8.9) (62)
	≥10 s	4.2 (2.5–6.6) (49)	8.4 (6.0–10.5) (67)	5.9 (4.0–9.6) (54)
	≥5 s	4.9 (2.8–8.0) (54)	9.4 (6.0–11.7) (74)	6.8 (4.5–11.8) (62)

**FIGURE 3:**
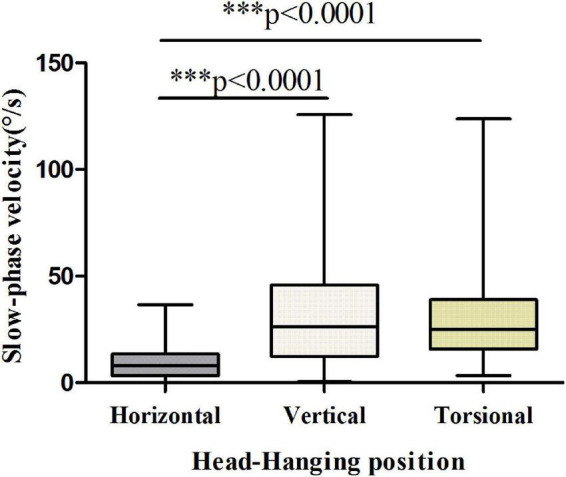
Slow-phase velocity of nystagmus in the head-hanging position of patients with PSC-can. The slow-phase velocity of nystagmus differed significantly between the horizontal and vertical components (*p* < 0.0001), horizontal and torsional components (*p* < 0.0001). *p < 0.05, ^**^p < 0.01, and ^***^p < 0.001, compared with respective control.

**FIGURE 4:**
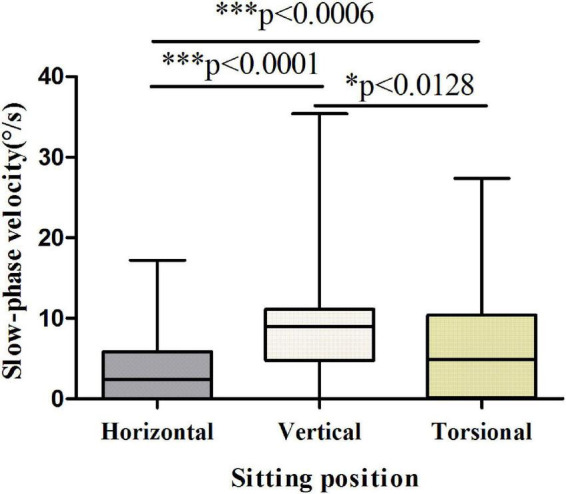
Slow-phase velocity of nystagmus in the sitting position of patients with PSC-can. The slow-phase velocity of nystagmus differed significantly between the horizontal and vertical components (*p* < 0.0001), horizontal and torsional components (*p* < 0.0006), vertical and torsional components (*p* < 0.0128). *p < 0.05, ^**^p < 0.01, and ^***^p < 0.001, compared with respective control.

The results showed that the vertical and torsional components of nystagmus induced by the Dix-Hallpike test were dominant in PSC-can patients, with the vertical component being the strongest (Figures 3, 4). The fast phase of the horizontal component was mostly contralateral in the head-hanging position, and the fast phase of the horizontal component after the return to the sitting position varied, being either ipsilateral or contralateral. According to the data, the average slow-phase velocity of the consecutive at 5 s was the strongest, so it is suggested that this slow-phase velocity should be used as a standard for quantitative analysis.

### Comparison between the head-hanging position and sitting position nystagmus of patients with PSC-can according to the Dix-Hallpike test

The ratio of latency between the head-hanging position and the sitting position was close to 2 (1.0–6.0) (*p* < 0.001), with the ratio of duration close to 1. The ratios of the slow-phase velocity of the horizontal, vertical, and torsion components of nystagmus for the head-hanging position and the sitting position were close to 1.85 (1.0–6.6), 3.7 (1.9–6.6), and 5.1 (2.6–11.3), respectively (Table 4).

**TABLE 4:** Comparison between the head-hanging position and sitting position nystagmus of 84 patients with PSC-can.

	Latency (s)			Duration (s)			Slow-phase velocity (°/s)		
	Horizontal	Vertical	Torsional	Horizontal	Vertical	Torsional	Horizontal	Vertical	Torsional
Head-hanging position	2.0 (1.0–3.5)	2.0 (1.0–4.0)	2.0 (1.0–3.5)	13.0 (10.0–19.0)	14.0 (10.0–19.0)	15.0 (12.0–21.0)	8.2 (3.2–12.4)	31.0 (12.8–47.4)	26.8 (16.0–39.3)
Sitting position	1.0 (0.0–1.0)	1.0 (0.5–2.0)	1.0 (0.5–1.0)	12.0 (0.0–19.0)	16.0 (10.0–22.0)	13.0 (5.0–18.0)	3.2 (0.0–6.3)	9.4 (5.6–11.6)	5.6 (2.8–10.7)
Head-hanging position/Sitting position	2 (1.0–6.0)	2 (1.0–5.0)	2 (1.0–5.0)	1.2 (0.8–10.0)	1.0 (0.7–1.4)	1.3 (0.9–5.2)	1.85 (1.0–6.6)	3.7 (1.9–6.6)	5.1 (2.6–11.3)
*p*-value	*p* < 0.001	*p* < 0.001	*p* < 0.001	*p* < 0.165	*p* < 0.966	*p* < 0.001	*p* < 0.001	*p* < 0.001	*p* < 0.001

### The ratio of slow-phase velocity in the head-hanging position and sitting position of patients with PSC-can according to the Dix-Hallpike test

The ratios of slow-phase velocity in the head-hanging position of the vertical to horizontal component, the torsional to horizontal component, and the vertical to torsional component were close to 3.3 (1.7–7.6), 3.9 (1.8–7.6), and 1.0 (0.5–1.8), respectively. The ratios of slow-phase velocity in the sitting position of the vertical to horizontal component, the torsional to horizontal component, and the vertical to torsional component were close to 2.1 (1.1–6.8), 1.5 (1.0–3.8), and 1.2 (0.8–2.8), respectively (Table 5).

**TABLE 5:** The ratio of slow-phase velocity in the head-hanging position and sitting position of 84 patients with PSC-can.

	Vertical/Horizontal	Torsional/Horizontal	Vertical/Torsional
Head-hanging position	3.3 (1.7–7.6)	3.9 (1.8–7.6)	1.0 (0.5–1.8)
Sitting position	2.1 (1.1–6.8)	1.5 (1.0–3.8)	1.2 (0.8–2.8)

## Discussion

According to Flourences and Ewald’s law, the fast phase of nystagmus induced in this study was in the same plane as that of the stimulated semicircular canal. The endolymph in the posterior semicircular canal flows away from the ampulla with excitatory stimulation (Dix and Hallpike, 1952; Honrubia and House, 2001). The posterior semicircular canal is at an angle of about 45° to the sagittal plane, and the position of the ampulla and the common crus were anteriorly inferior and posteriorly superior, respectively. Therefore, if the right posterior semicircular canal was excited under physiological conditions, it is necessary to turn the head to the left and upward. Then, a VOR synthesis vector is generated in which the slow phase eye movement rotates to the lower right, while the fast phase eye movement rotates to the upper left (the direction of head turning is the same as the direction of the induced nystagmus). In physiological state, the stimulation of the right posterior semicircular canal by turning the head to the left and upward was the same as the stimulation of the R-PSC-can in the right Dix-Hallpike test. In 3D-VNG, the horizontal, vertical and torsional components were to the left, upward and counterclockwise, respectively (Wang et al., 2009).

Compared with HSC-BPPV, PSC-BPPV objectively provides a physiological effect model to understand and study the “single semicircular canal stimulation response effect” under the action of “single factor,” but the study of PSC nystagmus mechanism and nystagmus characteristics has been one of the difficulties. In this study, the nystagmus characteristics of 84 patients with PSC-can were analyzed using 3D-VNG. With the patient in the hanging-head position of the Dix-Hallpike test in left ear and right ear PSC-can, the vertical components of nystagmus were upward, the torsional components were clockwise and counterclockwise, respectively, and the horizontal components were mainly contralateral. The median slow-phase velocity of the three components for consecutive 5 s was 26.3°/s (12.3–45.8), 25.0°/s (15.7–38.9) and 9.2°/s (4.9–13.7), respectively. When the patient was returned to the sitting position, the fast phase of the vertical and torsional components of nystagmus was reversed. In only one case the torsional component remained in the same direction. Among 54 patients with horizontal components of nystagmus, 32 patients the fast phase of the nystagmus remained unchanged. The median slow-phase velocity of the three components for consecutive 5 s was 9.4°/s (6.0–11.7), 6.8°/s (4.5–11.8), and 4.9°/s (2.8–8.0), respectively. The fast phase of the vertical and torsional components of nystagmus in patients with PSC-can was basically consistent with that in the BPPV guidelines, but information on the horizontal component of nystagmus was lacking in all of the guidelines. In this study, the horizontal component of nystagmus was not contralateral in 10 patients (13.7%) when the head was at the hanging-head position, and the direction of the horizontal component of nystagmus was not reversed in 32 patients (59.3%) when the head was at the sitting position. The reason leading to this may lies in two aspects: one is the coexistence of other types of vestibular terminal receptor dysfunction; the other is the variation of the anatomy and angle of each individual semicircular canal. For example, the posterior semicircular canal may not be completely perpendicular to the horizontal plane and has a horizontal Angle, resulting in horizontal components in different directions occur at the head-hanging position and sitting position. The 3D-VNG recorded and analyzed the nystagmus of PSC-can patients that was induced by the Dix-Hallpike test, which objectively showed the entirety fast phase of nystagmus, especially, the horizontal component of nystagmus is difficult to see with the naked eye, and more accurately showed the spatial relationship of the posterior semicircular canal plane.

Hiroaki’s (Ichijo, 2013) research results showed that Ewald’s third law is correct in the PSC, and the slow-phase velocity, the head-hanging position and those in the sitting position showed inversion asymmetry. The results of Zhang et al. (2021) showed the fast phase of nystagmus induced by HSC-can to be the same as the direction of head turning, and the slow-phase velocity ratio of the affected side to the healthy side was about 2:1. It has been reported that horizontal semicircular canal BPPV can be used as a single factor in the physiological effect model of a single horizontal semicircular canal stimulation response (Zhang et al., 2021), but no research report on the three-dimensional nystagmosis characteristics of P-BPPV based on this point of view was found. Xu’s (Xu et al., 2019) research results showed that the slow-phase velocity of vertical nystagmus induced by the head-hanging position on the affected side of the PSC-can was significantly greater than that induced by the sitting position, with the ratio of slow-phase velocity being about 2:1. However, the relationship of the slow-phase velocity between each component of PSC-can at two head positions and the relationship of the slow-phase velocity between the three components of each head position have not been reported (Hayashi et al., 2002; Aw et al., 2005; Imai et al., 2009; Eggers et al., 2019). Our results show that the ratios of the slow-phase velocity of the horizontal, vertical, and torsion components between the head-hanging position and sitting position were close to 1.85 (1.0–6.6), 3.7 (1.9–6.6) and 5.1 (2.6–11.3), respectively. The ratios of the slow-phase velocity of the vertical to horizontal component, the torsional to horizontal component, and the vertical to torsional component of the head-hanging position were close to 3.3 (1.7–7.6), 3.9 (1.8–7.6), and 1.0 (0.5–1.8), respectively. The ratios of the slow-phase velocity of the vertical to horizontal component, the torsional to horizontal component, and the vertical to torsional component of the sitting position were close to 2.1 (1.1–6.8), 1.5 (1.0–3.8), and 1.2 (0.8–2.8), respectively. These results suggest that PSC-can is the clinical manifestation of Ewald’s law in the human body. This study not only describes the orientation characteristics of the three components of nystagmus, but also further quantitatively analyzes each component. The results of this study further suggest that the three components of horizontal, vertical and torsional nystagmus induced by PSC-can are the result of the joint action of the PSC with the associated eye muscles dominated by ipsilateral superior/inferior oblique and contralateral superior/inferior rectus, but the weight of each group eye muscles and the interaction mechanism need to be further explored.

The limitation of this study is the absence of any control group. Therefore, normal people and PSC-can patients with unilateral and bilateral vestibular dysfunction should be included in subsequent studies for controlled studies. Because the PSC-can patients in this group did not have a comprehensive vestibular function evaluation, such as the ocular/cervical vestibular evoked myogenic potential (o/cVEMP) evaluation of otolithoid function and multifrequency functional testing of the three semicircular canals, the influence of otolithoid and other frequency functions of the three semicircular canals on nystagmus in PSC-can cannot be ruled out. There are other forms of the horizontal component of PSC-can nystagmus, and whether it can be used as an indication of other semicircular canal or otolith organ injury is worth further study in comprehensive vestibular function assessment. The 3D-VNG can only objectively record the characteristics of nystagmus. The diagnostic and differential diagnostic value of positional vertigo should be further verified by accumulating cases and increasing the time of observation and follow-up.

## Conclusion

In this study, 3D-VNG technology was applied to objectively record and analyze the direction, slow-phase velocity, and time parameter characteristics of each component of nystagmus in the head-hanging and sitting positions of patients with PSC-can in the Dix-Hallpike test. BPPV also objectively provides a physiological effect model to understand and study the “response effect of single posterior semicircular canal stimulation” under the action of “single factor on single posterior semicircular canal,” suggesting that PSC-Can is also the embodiment of Ewald’s law of human body. And can be used as a physiological stimulation model to understand the physiological function of the human posterior semicircular canal. In this study, the diagnosis and treatment of PSC-can was measured from naked eye observation to precise quantification, laying a foundation for future objective diagnosis, big data analysis and intelligent analysis of BPPV. At the same time, 3D-VNG can help to understand the mechanism and characteristics of PSC nystagmus.

## Data availability statement

The original contributions presented in this study are included in the article/supplementary material, further inquiries can be directed to the corresponding authors.

## Ethics statement

This study was reviewed and approved by the Ethics Committee of the Tianjin First Central Hospital (number: 2020N118KY). Written informed consent was obtained from the individual(s) for the publication of any potentially identifiable images or data included in this article.

## Author contributions

YL and TC wrote the main manuscript. XZ and QD collected the literature. QL, CW, and WW revised the manuscript. All authors reviewed the manuscript.
